# Model for small-angle scattering analysis of membranes with protein-like inclusions

**DOI:** 10.1107/S1600576725007277

**Published:** 2025-09-12

**Authors:** Cedric J. Gommes, Olga Matsarskaia, Julio M. Pusterla, Igor Graf von Westarp, Baohu Wu, Orsolya Czakkel, Andreas M. Stadler

**Affiliations:** a Department of Chemical Engineering, University of Liège B6A, Allée du Six Août 3, B-4000 Liège, Belgium; b Institut Laue–Langevin, 71 Avenue des Martyrs, 38042 Grenoble, France; c Jülich Centre for Neutron Science JCNS-1, Forschungszentrum Jülich GmbH, 52425 Jülich, Germany; d Institute of Physical Chemistry, RWTH Aachen University, Landoltweg 2, 52056 Aachen, Germany; e Jülich Centre for Neutron Science at MLZ, Forschungszentrum Jülich GmbH, Lichtenbergstrasse 1, 85747 Garching, Germany; Uppsala University, Sweden; The European Extreme Light Infrastructure, Czechia

**Keywords:** small-angle scattering, neutron spin–echo, membranes, proteins

## Abstract

Proteins are ubiquitous in biological membranes and have a significant impact on their scattering properties. In this contribution, we introduce a general mathematical construction to add proteins to any pre-existing membrane model and to calculate the resulting elastic and/or inelastic scattering cross section. The model is a low-resolution one, which describes the proteins as made up of regions of homogeneous scattering length density that extend through an arbitrary fraction of the membrane and possibly protrude out of it. In this construction, the protein characteristics that are relevant to scattering are their space and time correlation functions in the two-dimensional plane of the membrane. The results are particularized to a static bilayer model and to a Gaussian model of a fluctuating membrane. The models are then applied to the joint analysis of small-angle neutron and X-ray scattering of red blood cell membranes, of which transmembrane proteins constitute 25% of the volume, and to neutron spin–echo data measured on the same systems.

## Abstract 2

A general mathematical construction is proposed to add protein-like inclusions to any pre-existing membrane model and calculate the resulting small-angle scattering. The approach is suitable for both elastic and inelastic scattering data analysis.

## 1. Introduction

Membranes are central to biological systems, where they control interactions and exchanges between cells and their environment (Watson, 2015 ▸). Developing analytical tools to investigate their nanometre-scale structure and dynamics is central to understanding their physicochemical properties and how they fulfil their biological functions. Small-angle scattering of either X-rays or neutrons has a central role to play in that context, because it is one of the few experimental methods that can be used to investigate membranes on the nanometre scale under conditions close to those of their natural environment (Büldt *et al.*, 1978 ▸; Pusterla *et al.*, 2017 ▸; Pusterla *et al.*, 2020 ▸; Gommes *et al.*, 2021*a* ▸). Scattering methods, however, are often challenging because data analysis is required to convert reciprocal-space data into real-space structural or dynamic insights (Sivia, 2011 ▸; Squires, 2012 ▸). This step generally requires mathematical modelling (Pedersen, 1997 ▸; Gommes, 2018 ▸).

Many models are available for analysing the scattering from bilayer membranes, with different levels of structural sophistication (Kučerka *et al.*, 2004 ▸). In particular, models have been developed to capture the scattering resulting from uneven water distribution in various sections of the membrane (Kiselev *et al.*, 2008 ▸), from the lateral organization of the membrane and from bilayer asymmetry (Nickels *et al.*, 2015 ▸), from the curvature of the membrane (Chappa *et al.*, 2021 ▸), and from the random deformation of the membrane under the effect of thermal fluctuations (Gommes *et al.*, 2024 ▸).

An additional characteristic of biological membranes that contributes to making their scattering analysis challenging is the presence of membrane proteins. Such proteins are ubiquitous because they play central biological roles as chemical receptors, as controllers of molecular exchanges across the membrane, as enzymes and in many functions related to cellular adhesion (Mohandas & Gallagher, 2008 ▸; Himbert & Rheinstädter, 2022 ▸; Von Heijne, 2006 ▸; Kinnun *et al.*, 2023 ▸; Levental & Lyman, 2023 ▸; Krugmann *et al.*, 2020 ▸; Krugmann *et al.*, 2021 ▸). Red blood cells (RBCs) are excellent model systems for *in cellulo* studies using scattering techniques. In previous work, the properties of cytoplasmic water (Stadler *et al.*, 2008*b* ▸), haemoglobin diffusion and dynamics (Stadler *et al.*, 2008*a* ▸; Stadler *et al.*, 2011 ▸; Stadler *et al.*, 2012 ▸; Stadler *et al.*, 2014 ▸; Doster & Longeville, 2007 ▸; Longeville & Stingaciu, 2017 ▸), and haemoglobin–haemoglobin interactions in RBCs (Krueger & Nossal, 1988 ▸; Krueger *et al.*, 1990 ▸; Shou *et al.*, 2020 ▸) have been studied using neutron scattering methods. In the present work, we consider the scattering from vesicles consisting of RBC membranes. These membranes are complex systems consisting of asymmetric membranes with a heterogeneous lipid composition, including a large amount of cholesterol, and more than 50 types of transmembrane proteins (Mohandas & Gallagher, 2008 ▸; Himbert & Rheinstädter, 2022 ▸).

The presence of proteins has several different impacts on the scattering of membranes. The sheer presence of proteins reduces the contribution of the membrane to the scattering pattern and replaces it with a protein contribution. However, the relevant contrast is between the protein and the local scattering length density (SLD) of the membrane. A thorough analysis of the scattering therefore requires one to consider all the cross-correlations between the constituents of the membrane and of the protein. Recent modelling work testifies to this complexity (Anghel *et al.*, 2018 ▸; Spinozzi *et al.*, 2022 ▸; Spinozzi *et al.*, 2023 ▸).

In the present contribution, we introduce a general mathematical construction to add proteins to any pre-existing membrane model and to calculate the resulting elastic and/or inelastic scattering pattern. Because small-angle scattering is a low-resolution experimental method, the proteins are described here as regions with homogeneous SLD that cross the membrane and possibly protrude out of it. In addition to greatly simplifying the mathematics, this description is versatile enough to apply to a large variety of proteins and membranes. In this construction, the protein characteristics that are relevant to scattering are their external dimensions and their space and time correlation functions in the two-dimensional plane of the membrane.

The first section of the paper is experimental, focusing on the scattering data used to illustrate the models. They consist of small-angle neutron scattering (SANS) and small-angle X-ray scattering (SAXS) data measured on RBC membranes and of neutron spin–echo (NSE) data measured on the same systems. The general model is presented afterwards, and it is particularized to a static bilayer model and to a Gaussian model of a fluctuating membrane. These models are then used in the *Discussion*[Sec sec4] section to analyse the elastic and inelastic scattering data measured on the RBC membranes.

## 2. Experimental

RBC liposomes were obtained from the blood of healthy anonymous volunteers, provided by the French Blood Bank (Établissement Français du Sang, Grenoble), for neutron scattering experiments at the Institut Laue–Langevin (ILL) in Grenoble, France. Fresh blood was taken from healthy human volunteers by venipuncture to produce RBC liposomes for SAXS experiments at the Heinz Maier-Leibnitz Zentrum (MLZ) in Garching, Germany. Blood was stored at 4°C prior to sample preparation.

The preparation of RBC liposomes followed a slightly modified protocol as initially described by Himbert *et al.* (2017 ▸, 2022 ▸). All used chemicals were obtained from Sigma–Aldrich (Massachusetts, USA). The RBC samples were centrifuged at 2000 relative centrifugal force (rcf) for 5 min at 4°C to spin down the RBC pellet. The supernatant consisting of blood plasma including the ‘buffy coat’ was removed, and the RBC pellet was washed with 300 mOsm phosphate-buffered saline (PBS) buffer (containing 137 m*M* NaCl, 2.7 m*M* KCl, 10 m*M* Na_2_HPO_4_, 1.8 m*M* KH_2_PO_4_ and 10 m*M* glucose). The washing procedure was performed three times in total. The RBC pellet was subsequently suspended in pre-chilled lysis buffer (3% PBS buffer pH 8.0) to final RBC volume fractions of around 20% for the SANS and NSE experiments and 7% for the SAXS measurement. The suspension was vortexed for 10 s, incubated on ice for 30 min, and centrifuged at 15600 rcf (SANS), 30000 rcf (SAXS) or 60000 rcf (NSE) for 30 min at 5°C to spin down the RBC membrane fraction. Finally, the RBC membranes were washed four times with 300 mOsm PBS buffer by centrifuging them for 30 min at 5°C. The last three washing steps in preparation for the neutron scattering experiments were done with D_2_O-based PBS buffer (99.9% atom D). The RBC membranes were then diluted to nominal lipid concentrations of 6 mg ml^−1^ for the SAXS measurement and 30 mg ml^−1^ for the SANS and NSE experiments.

To obtain RBC liposomes, these RBC membrane solutions were sonicated on ice using a tip sonicator. To this end, 20 repetitions of 5 s pulses at a power of 75 W (SANS and NSE) or 100 W (SAXS) were performed, followed by a cooling phase of 25 s. The RBC liposome solutions were then finally centrifuged at 20000 rcf for 30 min, which allows separation of the unilamellar RBC liposomes from larger aggregated particles and larger multilamellar vesicles (Himbert *et al.*, 2017 ▸). A fraction of the 30 mg ml^−1^ RBC liposome solution was diluted to 3 mg ml^−1^. This procedure allowed us to obtain RBC liposome solutions at nominal concentrations of 6 mg ml^−1^ for SAXS, 3 and 30 mg ml^−1^ for SANS, and 30 mg ml^−1^ for NSE experiments.

SAXS measurements were performed on the laboratory instrument KWS-X (Xeuss 3.0, Xenocs, Grenoble, France) at the MLZ. The samples were filled into glass capillaries with a diameter of 2 mm. Measurements were performed at sample-to-detector distances of 0.5 and 1.7 m using an X-ray wavelength of 1.34 Å, corresponding to a *q* range of 0.0044–1.177 Å^−1^. The KWS-X instrument uses a MetalJet D2+ as X-ray source (Excillum, Kista, Sweden). Two-dimensional scattering data were recorded on a moveable Eiger2 R 4M detector at ambient temperature and reduced to 1D patterns using the *XSACT* software (as provided by Xenocs). Scattering intensities were corrected for transmission and for solvent scattering. The modulus of the scattering vector **q** is defined in this work as 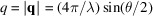, with the incident X-ray or neutron wavelength λ and the scattering angle θ.

SANS measurements were performed on D22 at the ILL (Matsarskaia *et al.*, 2023 ▸). Samples were measured in rectangular quartz cells (Aireka Scientific, Hong Kong, China) with 1 mm thickness. All measurements were performed at 21°C. A *q* range of 0.005–0.642 Å^−1^ was covered using a wavelength of 6 Å and a collimation and sample-to-detector distance of 17.6 m. Raw data were corrected using the scattering of the empty sample cell and the electronic noise (via measuring B_4_C). Normalization to absolute scattering intensity was done using an attenuated empty beam measurement. Reduction of the 2D detector images to 1D spectra was performed using the software *Grasp* (Dewhurst, 2023 ▸). Prior to further data analysis, the PBS D_2_O buffer signal was subtracted from the RBC liposome sample signal. RBC liposomes were measured at nominal concentrations of 3 and 30 mg ml^−1^ with SANS. After division by the nominal RBC liposome concentration, the SANS data of the 3 and 30 mg ml^−1^ solutions overlapped within the statistical uncertainty, and no structure factor effects were observed in the SANS data of the 30 mg ml^−1^ RBC liposome solution. Hence, the experimental SANS data of the 30 mg ml^−1^ liposome solutions were used for further data analysis as described below.

**Figure 1:**
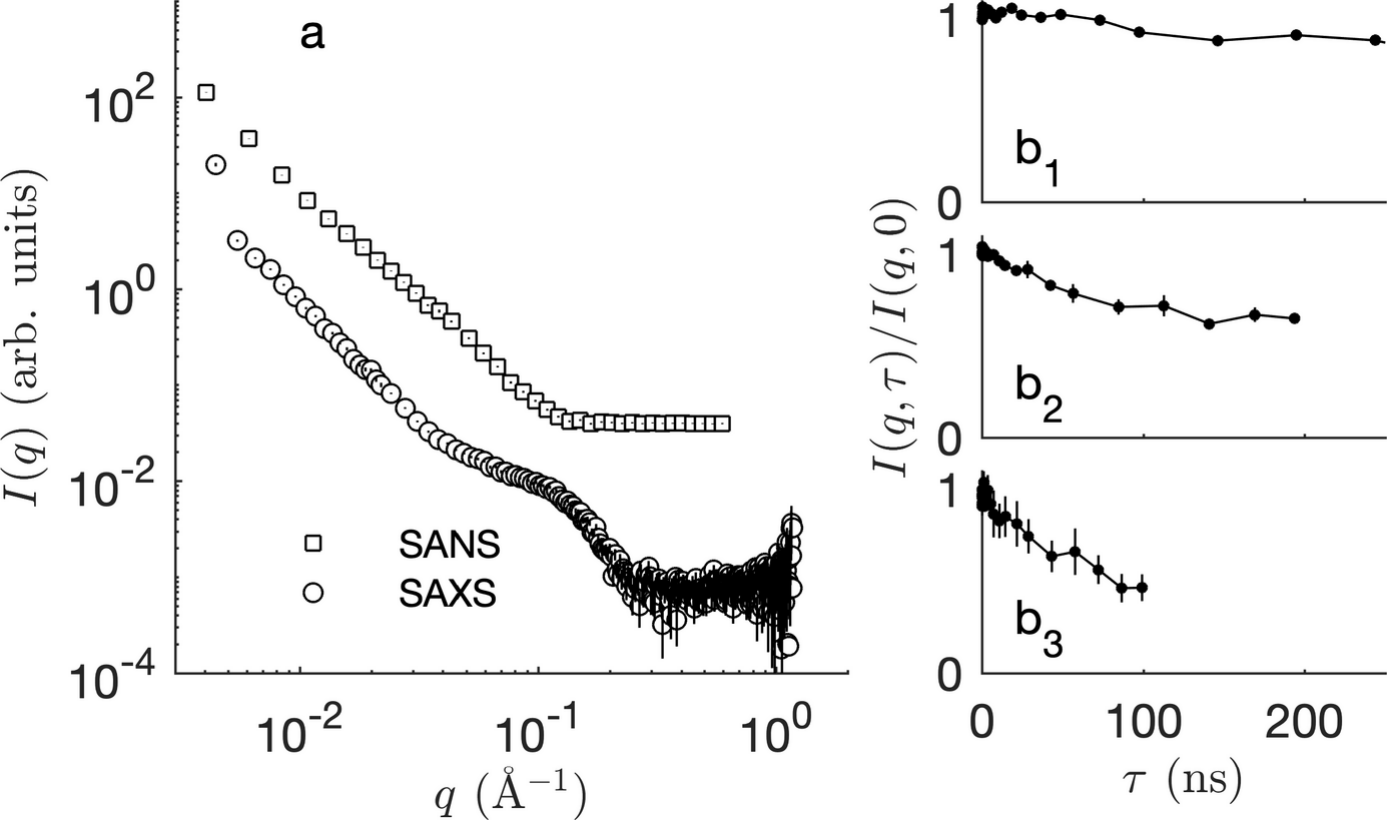
(*a*) SANS and SAXS scattering by RBC membranes and (*b*) NSE data on the same systems at (*b*_1_) *q* = 0.036 Å^−1^, (*b*_2_) *q* = 0.071 Å^−1^ and (*b*_3_) *q* = 0.109 Å^−1^.

The SANS and SAXS patterns of the 30 and 6 mg ml^−1^ RBC liposome solutions, respectively, are shown in Fig. 1 ▸(*a*). They are typical of membrane scattering patterns, with an overall *q*^−2^ scattering at low *q* pointing to the overall 2D structure of the membrane. The deviations from this trend at higher *q* characterize the inner structure of the membrane.

To capture the dynamic properties of the RBC membranes, NSE measurements were performed on the IN15 instrument at the ILL (Matsarskaia *et al.*, 2023 ▸). Samples were filled into 2 mm quartz cells (Hellma, Müllheim, Germany). Three incident neutron wavelengths of 8, 10 and 12 Å were used during the experiment. The instrumental resolution was measured for each setup using the elastic scattering of graphite. The NSE data are expressed in terms of the intermediate scattering function *I*(*q*, τ) as a function of both the modulus of the scattering vector *q* and the correlation time τ (Squires, 2012 ▸). Elastic scattering (*e.g.* SAXS or SANS) is a particular case of *I*(*q*, τ) for τ = 0. The obtained *I*(*q*, τ)/*I*(*q*, 0) were corrected for transmission and buffer scattering. The normalized data *I*(*q*, τ)/*I*(*q*, 0) are shown in Fig. 1 ▸(*b*) for three representative values of *q*.

## 3. Modelling

### 3.1. General formalism

We consider here a general formalism which encompasses both elastic and inelastic scattering. Consequently, the system is described through its space- and time-dependent SLD ρ(**x**, *t*) at a point **x** and time *t*. The intermediate scattering function is then expressed as the following Fourier transform (Van Hove, 1954 ▸; Squires, 2012 ▸; Gommes *et al.*, 2021*b* ▸): 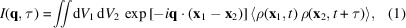
where each integral is over the entire space. Here and throughout the paper, angle brackets 〈 〉 stand for a time average or an ensemble average. In the case of deterministic and time-independent models, the brackets can simply be ignored. The classical expression for the scattering cross section for elastic scattering (SAXS or SANS) is obtained as a particular case of equation (1) by setting τ = 0. Finite values of τ are notably relevant to the NSE signal, which is generally reported as *I*(*q*, τ)/*I*(*q*, 0).

In the specific case of a membrane, it is convenient to express the intermediate scattering function *I*(**q**, τ) per unit area *A* of the projected membrane. The thus-defined intermediate scattering function is then the classical Fourier transform over a variable **r** of the following correlation function: 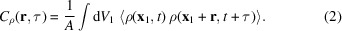
If the membrane model is statistically stationary in its plane, all statistical properties are translation invariant in the plane. In other words, the **x**_1_ dependence of the integrand is only through its component orthogonal to the membrane, say along *z*. We can therefore write 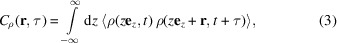
where **e**_*z*_ is a unit vector along *z*.

For further purposes, it is convenient to consider explicitly the case of a membrane made up of several layers, each with a distinct SLD 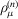. In that case the overall SLD can be written as 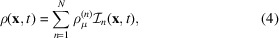
where 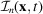 is the indicator function of the *n*th layer, equal to 1 if point **x** is in layer *n* at time *t* and to 0 otherwise. In the context of scattering by a randomly fluctuating membrane under the effect of thermal fluctuations, it is convenient to use a probabilistic interpretation of the angle brackets 〈 〉 in equation (1) and to define the following two-point probability function:
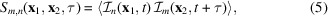
corresponding to the probability of points **x**_1_ and **x**_2_ belonging to the *n*th and *m*th layers at different times separated by an interval τ. With that notation, the correlation function of a membrane with various sublayers, as defined in equation (3), takes the form 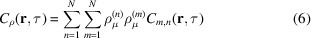
with 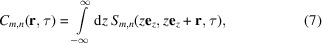
where the integral with respect to *z* is over the entire thickness of the membrane. The elastic or inelastic scattering cross section of the membrane is then obtained as the Fourier transform of equation (6) on variable **r**.

**Figure 2:**
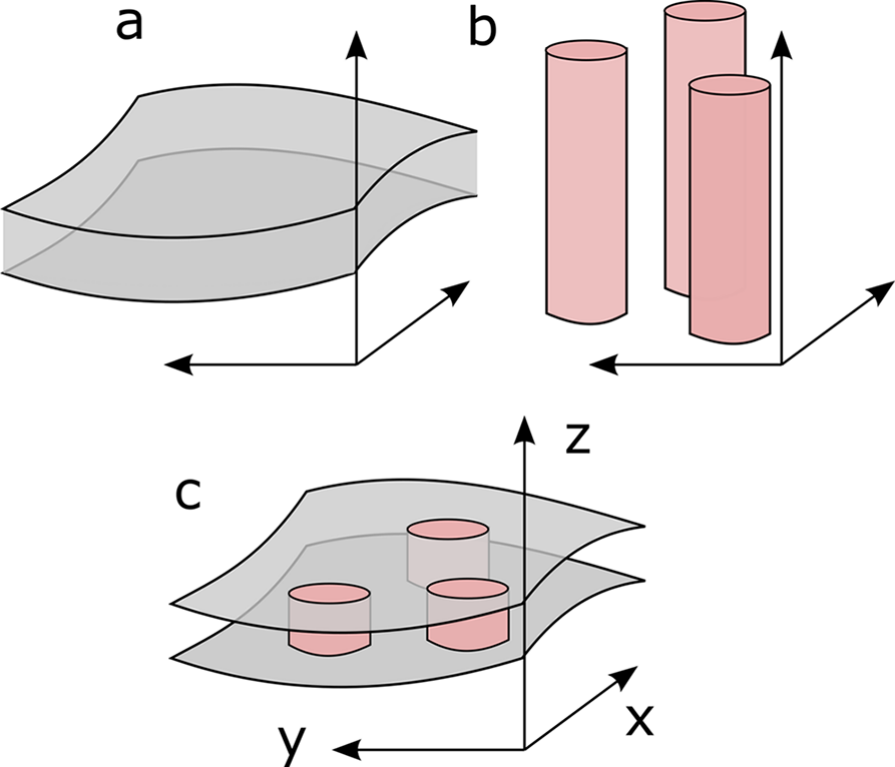
General modelling construction with independent (*a*) membrane and (*b*) protein models, which are intersected to create (*c*) the membrane with included proteins.

The general approach that we propose to model fluctuating membranes with included proteins is based on the construction sketched in Fig. 2 ▸. In that construction, the membrane model [Fig. 2 ▸(*a*)] is complemented by an *independent* 2D model in the *xy* plane to describe the position and extent of proteins in the membrane plane [Fig. 2 ▸(*b*)]. The latter 2D structure is extended in the *z* direction as infinite cylinders, which are then intersected with the membrane model. In the sketch of Fig. 2 ▸ the cylinders are shown with circular cross sections, but there is no restriction on the possible shape of the cross sections.

From a mathematical perspective, the construction in Fig. 2 ▸ boils down to modelling the SLD as 
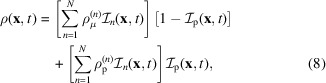
where 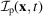 is the indicator function of the cylinder associated with the proteins and 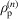 is the local SLD of the protein within the *n*th layer of the membrane. Because indicator functions can only take the value 0 or 1, the first term in equation (8) assigns the SLD of the membrane 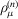 to the points in space outside the cylinders and the second term assigns the SLD of the protein 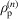 to the points inside the cylinders.

**Figure 3:**
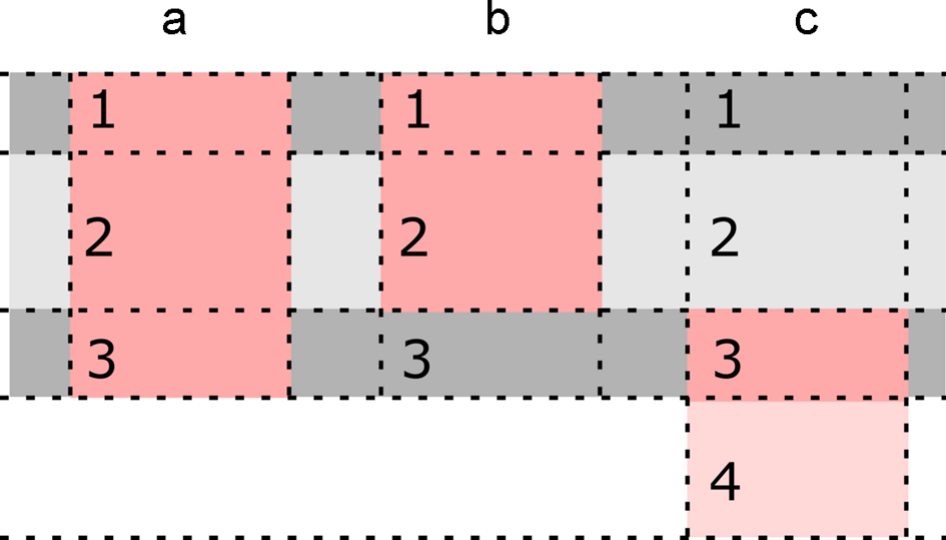
Examples of configurations captured by equation (8), whereby the protein (*a*) crosses through the membrane, (*b*) extends through a fraction of the membrane or (*c*) protrudes out of the membrane. In the last case, a bogus layer is added to the membrane with the same SLD as the solvent. The different colours in the figure denote different SLDs.

Note that the general expression in equation (8) also allows us to address the case of proteins protruding from the membrane, possibly in an asymmetric way (see Fig. 3 ▸). This situation is modelled by adding bogus layers on both sides of the true membrane with the same SLD as the solvent, *e.g.*
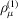
 = 
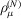
 = 0. Letting the protein SLD be *n* dependent enables one to assign a lower scattering contrast to any protruding part, as would be expected if these parts were swollen with solvent. Similarly, the approach also enables one to model a protein that extends through an arbitrary fraction of the membrane, by equating the SLDs 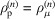 for suitable values of *n*.

Starting from equation (1), and without any assumption besides the statistical independence of the protein and membrane structures, *i.e.* of 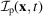 and 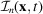, one finds that the correlation function that replaces equation (6) in the case of membranes with included proteins is 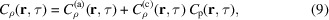
with 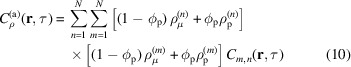
and 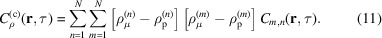
Here, ϕ_p_ is the volume fraction of the protein cylinder phase, which is equal to the volume fraction of proteins within the membrane in the case of Fig. 3 ▸(*a*). Comparison with equation (6) shows that 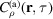 is the correlation function of the original membrane with the SLD of each layer 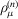 replaced by an *average* value that accounts for the presence of proteins 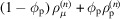. The correlation function 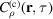 has a similar interpretation, with the SLD of each layer 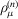 replaced by its *contrast* with the protein 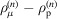. The last factor in equation (9) is the correlation function of the protein cylinder phase, defined as 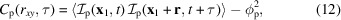
where the angle brackets are ensemble averages or averages over **x**_1_. Note that, by construction of the cylinders, the dependence of *C*_p_(**r**, τ) on **r** is only through its component in the plane *r*_*xy*_. The last term in equation (12) ensures that the correlation function *C*_p_(**r**, τ) converges to 0 for large values of *r*_*xy*_.

### 3.2. Specific models

#### 3.2.1. Protein model

We are concerned here with the modelling of the cylinder phase in Fig. 2 ▸(*b*), which aims to capture the spatial distribution of proteins within the membrane plane. Mathematically, this boils down to proposing an analytical expression for *C*_p_(**r**, τ) in equation (9), which is independent of the membrane model itself.

In the specific case of elastic scattering, the relevant correlation function is *C*_p_(**r**, 0), *i.e.* for τ = 0. In that case, a possible approach consists of assuming a (2D) monodispersed hard-disc model with radius *R*_p_. This is equivalent to writing the 2D Fourier transform of *C*_p_(**r**, 0), 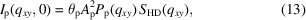
where θ_p_ is the number of proteins per unit area of the *xy* plane, 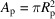 is their projected area and *P*_p_(*q*_*xy*_) is their form factor. 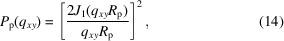
where *J*_1_( ) is the Bessel function of the first kind of order 1. *S*_HD_(*q*_*xy*_) is the hard-disc structure factor. There exists no exact analytical form for *S*_HD_(*q*_*xy*_), but the following is a good approximation for low densities (Baus & Colot, 1986 ▸; Studart *et al.*, 1996 ▸): 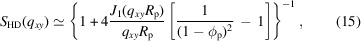
where ϕ_p_ = θ_p_*A*_p_ is the volume fraction of protein cylinders.

Although this approach based on a structure factor can in principle be generalized to inelastic scattering by time-dependent systems (Zhang *et al.*, 2014 ▸), this can be done more easily with Boolean models (Sonntag *et al.*, 1981 ▸; Jeulin, 2000 ▸; Gommes, 2022 ▸). In that spirit, the interactions of the proteins are ignored altogether and the discs are allowed to overlap. Due to this overlap, the volume fraction of the protein does not depend linearly on their surface concentration θ_p_ and the relationship is 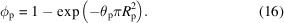
For ϕ_p_ ≃ 0.25 the overlapping of discs amounts to less than 4% of the space. Although letting the discs (and proteins) overlap is physically unrealistic, from a scattering perspective this assumption is relatively inconsequential. The corresponding in-plane correlation function is 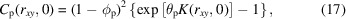
where *K*(*r*_*xy*_, 0) is the intersection area of two discs with their centres at a distance *r*_*xy*_ from each other, namely 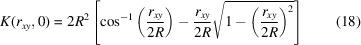
for *r*_*xy*_ ≤ 2*R* and *K* = 0 otherwise. In the limit of 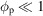, the correlation function converges to *C*_p_ ≃ θ_p_*K*. In that limit, the scattering coincides with equation (13) with *S*_HD_ = 1. The approach based on Boolean models is easily generalized to inelastic scattering from time-dependent structures by letting the proteins move in the *xy* plane. Equation (17) remains valid in that general case, only with *K*(*r*_*xy*_, τ) obtained from *K*(*r*_*xy*_, 0) in equation (18) via convolution with the protein displacement law (Gommes, 2022 ▸).

#### 3.2.2. Slab membrane model

The simplest membrane model we consider is a static and deterministic scattering density profile ρ(*z*), independent of both *x* and *y* (Kiselev *et al.*, 2002 ▸; Kučerka *et al.*, 2004 ▸). In this case, the Fourier transform of *C*_ρ_(**r**) from equation (3) is 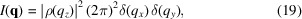
where ρ(*q*_*z*_) is the 1D Fourier transform of ρ(*z*) and the two delta functions arise from *C*_ρ_(**r**) being independent of the in-plane components of **r**. The rotational average of *I*(**q**) uniformly over all directions of space is 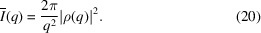
This results from integrating equation (19) over a sphere of radius *q* and dividing by its area 4π*q*^2^.

When proteins are present in the membrane, the corresponding scattering is obtained as the Fourier transform of equation (9). After averaging over all directions of space, the result is 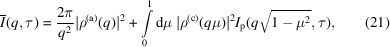
where ρ^(a)^(*q*_*z*_) and ρ^(c)^(*q*_*z*_) are the Fourier transforms of the modified SLD profiles with ρ(*z*) → (1 − ϕ_p_)ρ(*z*) + ϕ_p_ρ_p_ and ρ(*z*) → ρ(*z*) − ρ_p_, respectively. In the second term of equation (21) the integration results from rotational averaging. μ is the cosine of the azimuthal angle and *I*_p_(*q*, τ) is the 2D Fourier transform of *C*_p_(*r*_*xy*_, τ) within the membrane plane, namely 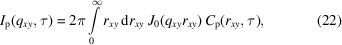
where *J*_0_( ) is the Bessel function of the first kind of order zero. Note that, in the particular case of elastic scattering by a static structure, *I*_p_(*q*_*xy*_, 0) can be modelled as in equation (13).

We now consider the particular case of membranes made up of amphiphilic molecules with a hydrophobic chain of length *l*_C_ and a hydrophilic head of thickness *l*_H_. To keep the discussion general, we also allow the presence of solvated parts of proteins that protrude from the membrane over a distance *l*_S_ on both sides. The Fourier transform of the corresponding SLD is 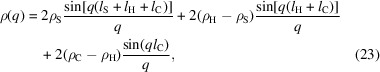
where ρ_S_, ρ_H_ and ρ_C_ are the SLDs in each region. This expression corresponds to the scattering density contrasted with the void. When contrasted with the solvent and in the presence of membrane proteins, ρ_S_, ρ_H_ and ρ_C_ must first be formally replaced by the protein-corrected average and contrasted values, from which ρ_S_ is afterwards subtracted. In other words, ρ^(a)^(*q*) and ρ^(c)^(*q*) are obtained by replacing the SLDs in equation (23) by 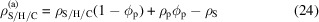
and 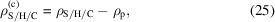
respectively.

#### 3.2.3. Gaussian membrane model

**Figure 4:**
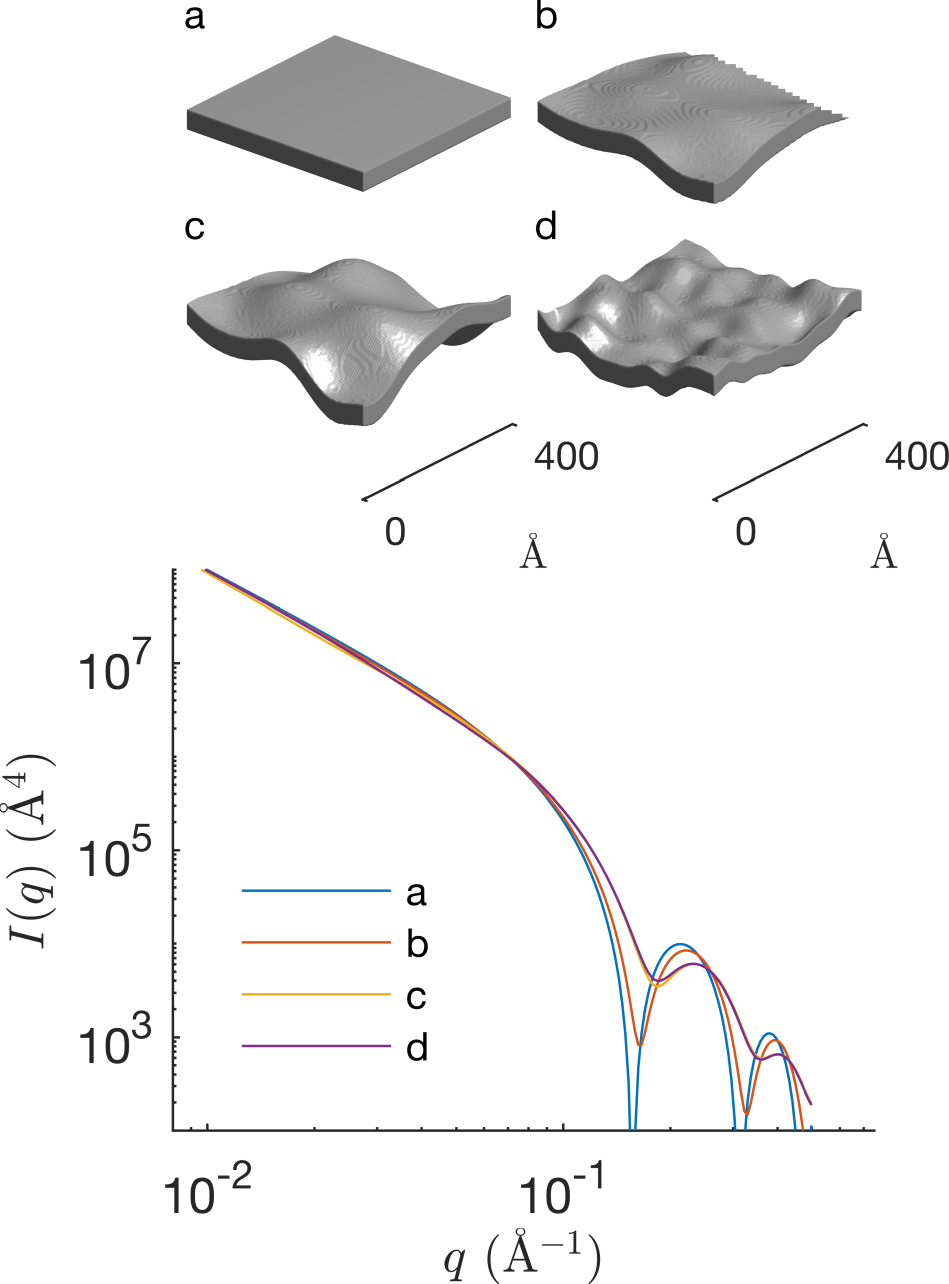
Realizations of the Gaussian membrane model with thickness 40 Å and (*a*) *l*_α_ = 0, identical to a slab model, (*b*) *l*_α_ = 15 Å and *l*_*xy*_ = 100 Å, (*c*) *l*_α_ = 30 Å and *l*_*xy*_ = 100 Å, and (*d*) *l*_α_ = 15 Å and *l*_*xy*_ = 50 Å. The corresponding scattering patterns for a homogeneous SLD are shown underneath.

The general procedure in Fig. 2 ▸ and equation (9) for adding proteins to an existing membrane model is not restricted to slabs. We discuss here the case of a Gaussian membrane model, which was developed for the joint analysis of both elastic and inelastic scattering data (Gommes *et al.*, 2024 ▸). The model is illustrated in Fig. 4 ▸. In addition to the same parameters as in the slab models, capturing the thicknesses of the various sublayers, the Gaussian model has other parameters characterizing the random fluctuations.

In the general model, both compression and bending fluctuations are accounted for. In the simpler version that we consider here, only bending fluctuations are included. These are captured by two parameters: a characteristic length *l*_α_ controlling the amplitude of the fluctuations, and a characteristic length *l*_*xy*_ controlling the size of the deformations in the direction parallel to the membrane. The different effects of these two parameters are illustrated in Figs. 4 ▸(*a*) to 4 ▸(*d*). In the case of inelastic scattering, additional parameters are needed to characterize the dynamics of the fluctuations, as we will discuss later.

In the context of the Gaussian model, the correlation function *C*_ρ_(**r**, τ) comprises two contributions, 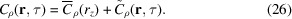
The first term accounts for the average structure, averaged either over time or over the entire *xy* plane. The second term accounts for the deviations from the average, *i.e.* for the statistical fluctuations, which are generally time dependent. Mathematically, the first term in equation (26) is the self-convolution of the average SLD profile, namely 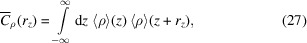
where 〈ρ〉(*z*) is given in equation (22) of Gommes *et al.* (2024 ▸). Because this function depends only on the coordinate *r*_*z*_, it leads to a contribution identical to the slab model in equation (21).

The second term in equation (26) characterizes the deviations from the average structure. The latter fluctuations are captured in the model by a Gaussian random field, which is comprehensively described by its space and time correlation function. The specific analytical form that we assume here is 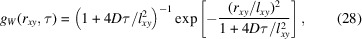
which is a particular case of equation (45) of Gommes *et al.* (2024 ▸) in the limit *l*_*z*_ → ∞. This limit is structurally equivalent to suppressing compression fluctuations of the membrane. Equation (28) describes the membrane bending in terms of Gaussian wave packets of size *l*_*xy*_ that randomly move in the *xy* plane with diffusion coefficient *D* and randomly overlap positively or negatively. Based on *g*_*W*_(*r*_*xy*_, τ), the scattering length correlation function 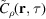 is calculated using equation (26) of Gommes *et al.* (2024 ▸). The latter equation still holds when proteins are present in the membrane, but two versions are calculated for the protein-corrected SLDs 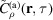 and 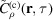 in line with equations (10) and (11).

Note that the correlation function of the fluctuations 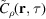 depends on both the out-of-plane *r*_*z*_ and in-plane *r*_*xy*_ components of **r**. In that case, unlike the slab model, the evaluation of the second term in equation (9) cannot be done directly in reciprocal space. Therefore, calculating the protein scattering with the Gaussian membrane model requires an analytical expression for the protein correlation function *C*_p_(*r*_*xy*_, τ) in real space. In that respect, the Boolean model is more convenient than the hard-disc model [see equation (17)].

## 4. Discussion

The chemical composition of RBC membranes is notoriously complex, with a variety of lipid molecules forming a heterogeneous bilayer structure containing both ordered and disordered phases, in which up to 50 different types of proteins are embedded (Mohandas & Gallagher, 2008 ▸; Himbert & Rheinstädter, 2022 ▸). In order to analyse the scattering data in Fig. 1 ▸ with as simple a model as possible, we assume a unique average lipid comprising a hydrophilic head and hydrophobic chain with thicknesses *l*_H_ and *l*_C_, respectively. Biological RBCs also have a spectrin-based cytoskeleton, which is anchored to the RBC membrane via the junctional and ankyrin protein complexes (Bennett & Baines, 2001 ▸). As a consequence of the specific RBC liposome preparation used in this work, the cytoskeleton is absent from the samples, but the membrane proteins are largely preserved.

Dupuy & Engelman (2008 ▸) provide an estimate of 23% for the overall transmembrane fraction of RBC. Himbert *et al.* (2017 ▸) report that these proteins can effectively be represented as domains having a characteristic size of 28 Å in the membrane plane and a transmembrane thickness of 40.6 Å. More detailed analysis is obtained by separating the proteins using gel electrophoresis; the prevailing proteins are visible as a third band on such gels. The biological function of these ‘band 3’ proteins is to transport anions across the membrane (Aoki, 2017 ▸; Poole, 2000 ▸). Because of their prevalence, we hereafter consider that the band 3 protein is representative of all other RBC membrane proteins.

**Figure 5:**
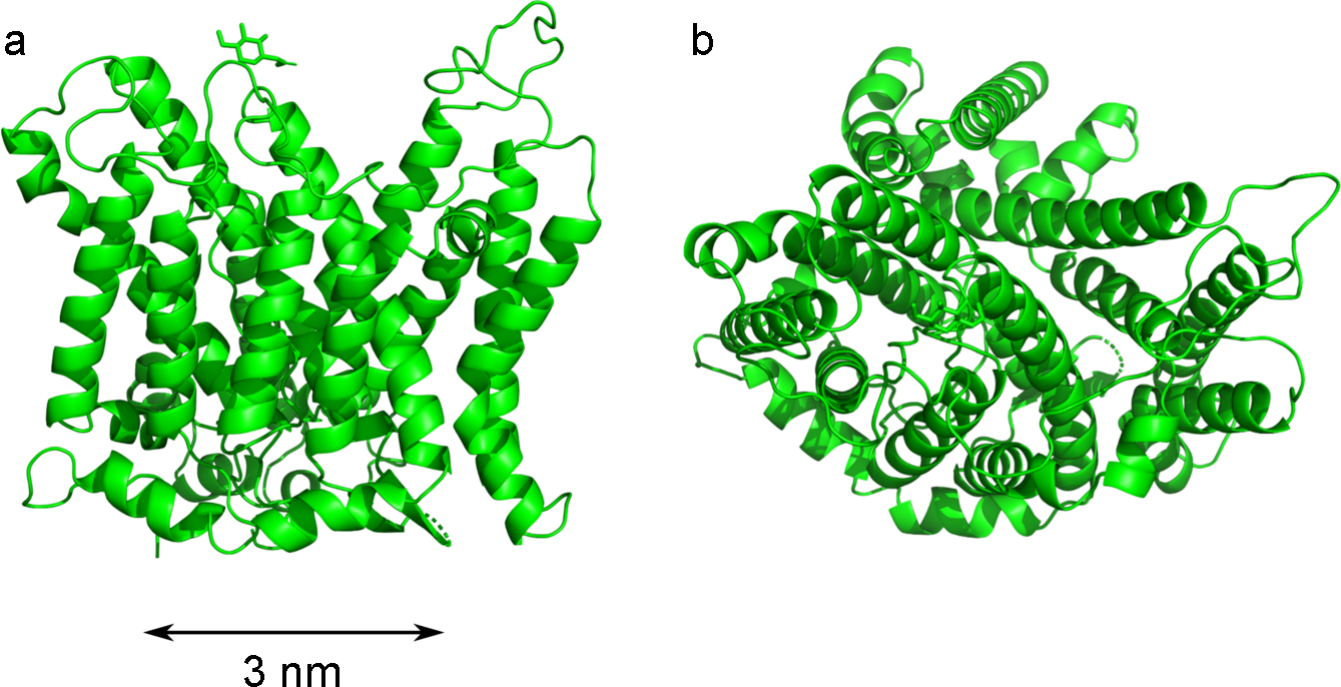
Structure and size of the membrane component of the band 3 protein in the monomeric state as found in RBC membranes, as determined by cryo-electron microscopy. (*a*) Side view and (*b*) top view.

The approximate structure and size of the band 3 protein have been determined using *AlphaFold* (Abramson *et al.*, 2024 ▸). The *AlphaFold* protein model has a thickness of around 40 Å, close to the thickness of the RBC membrane, and a comparable size in the orthogonal direction. Independent cryo-electron microscopy (cryo-EM) shows that the shape of this band 3 protein as a monomer is that of an ellipse with major/minor axes of 59/31 Å and a transmembrane length of 47 Å (PDB ID 7tw2; Xia *et al.*, 2022 ▸). These dimensions are similar to those reported by Himbert *et al.* (2017 ▸), which reassures us in our assumption that the band 3 protein is representative of all transmembrane domains in RBCs. The cryo-EM structure (Xia *et al.*, 2022 ▸) is shown in Fig. 5 ▸. The structural models shown in the figure were generated using *PyMOL* (Schrödinger LLC, 2015 ▸). In the range of *q* relevant to the data of Fig. 1 ▸, the inner structure of the band 3 protein cannot be resolved by small-angle scattering. In order to calculate the scattered intensities, the band 3 protein is therefore assumed to have a homogeneous SLD. The following values are calculated from the protein composition: ρ_p_ ≃ 12.062 × 10^−6^ Å^−2^ for X-rays and ρ_p_ ≃ 1.685 × 10^−6^ Å^−2^ for neutrons (Kienzle, 2025 ▸).

**Figure 6:**
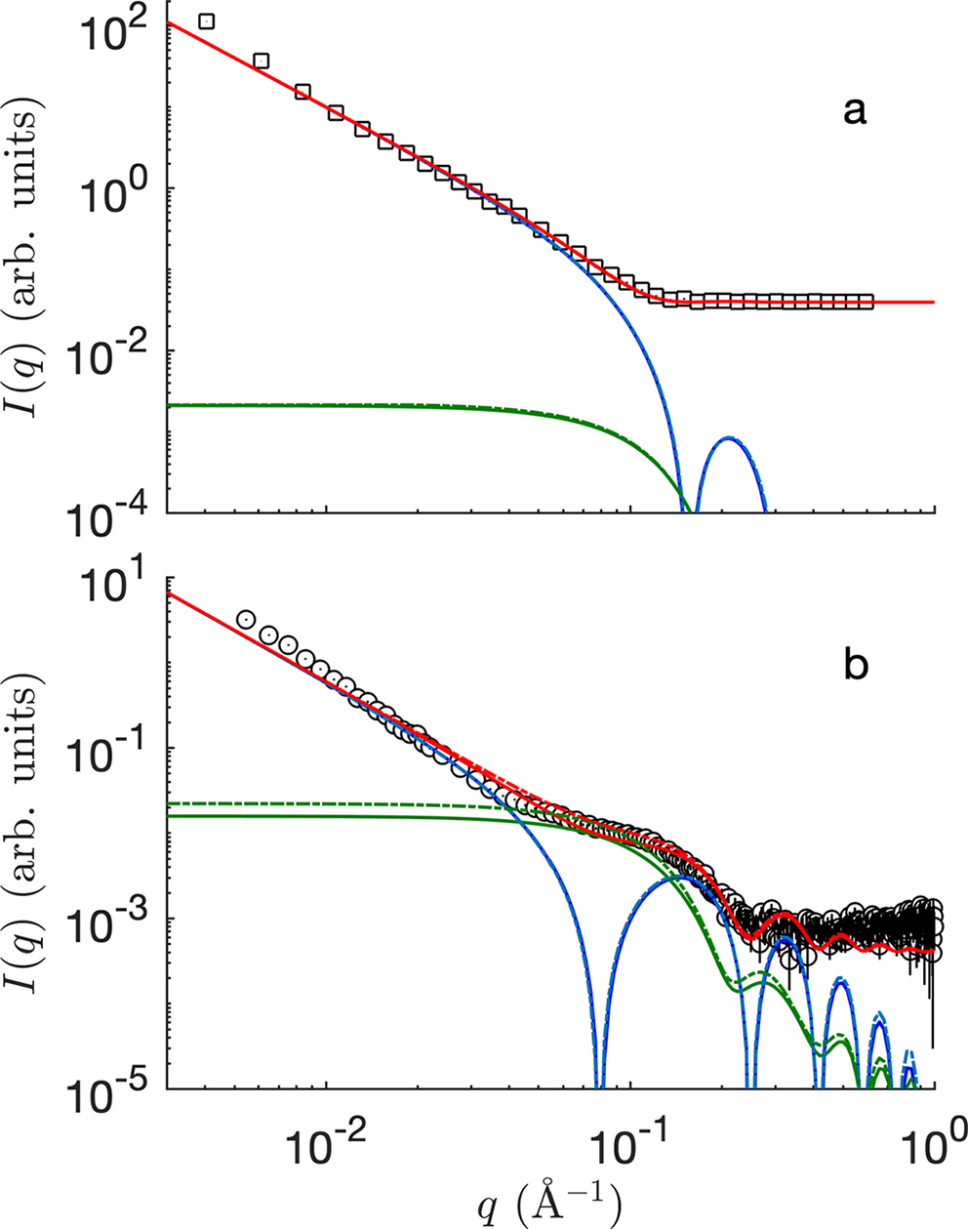
Fitting of the membrane (*a*) SANS and (*b*) SAXS data with the slab model with included proteins. The blue and green lines are the membrane and protein contributions, respectively, corresponding to the first and second terms in equation (21), and the red curve is their sum. The solid and dashed lines are the Boolean and hard-disc protein models, respectively. The fitting range extends from *q* = 8 × 10^−3^ Å^−1^ to *q* = 0.5 Å^−1^.

The fitting of the membrane scattering data by the slab model with included proteins is shown in Fig. 6 ▸. The band 3 protein is modelled as an equivalent cylinder with radius *R*_p_ = 21.4 Å, corresponding to an area 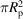 identical to that of the actual elliptical section. The protein is assumed not to extend beyond the limits of the membrane, corresponding to setting *l*_S_ = 0 in equation (23). The following SLDs are used for the lipid heads and chains: ρ_H_ ≃ 14.2 × 10^−6^ Å^−2^ and ρ_C_ ≃ 8.1 × 10^−6^ Å^−2^ for X-rays, and ρ_H_ ≃ 1.87 × 10^−6^ Å^−2^ and ρ_C_ ≃ −0.07 × 10^−6^ Å^−2^ for neutrons, which are borrowed from earlier work (Gommes *et al.*, 2024 ▸). These values are comparable (though not identical) to the values calculated for complex RBC membranes according to the composition reported by Himbert *et al.* (2021 ▸). In all cases, the solvent is heavy water, with ρ_W_ ≃ 9.37 × 10^−6^ Å^−2^ for X-rays and ρ_W_ ≃ 6.37 × 10^−6^ Å^−2^ for neutrons.

Because the radius of the protein/cylinder is imposed, the only fitting parameters for the fit are *l*_H_ and *l*_C_. Both the hard-disc and Boolean models were tested, and the fitted parameters are very similar in the two cases: *l*_C_ ≃ 16.3 Å and *l*_H_ ≃ 5.0 Å for the Boolean model (total membrane thickness 42.6 Å), and *l*_C_ ≃ 16.6 Å and *l*_H_ ≃ 4.5 Å for the hard-disc model (total membrane thickness 42.2 Å). In the case of the SANS data, the two models are indistinguishable on the scale of Fig. 6 ▸. Note that the total thickness of the membrane is close to the size of the band 3 protein in that direction [Fig. 5 ▸(*a*)], which retrospectively justifies our assumption that the protein does not protrude from the membrane.

In the case of neutron SLDs, the protein contribution to the scattering is found to be two orders of magnitude smaller than the membrane contribution [Fig. 6 ▸(*a*)]. This means that the contribution of band 3 to the SANS is comprehensively captured by its effect on the average SLD, *i.e.* by the first term in equation (9). Interestingly, this is not the case for the SAXS, for which the protein itself is the the largest contributer to the scattering at intermediate *q*. In particular, the second term in equation (9) is responsible for the shoulder in the scattering pattern around *q* ≃ 0.1 Å^−1^ [Fig. 6 ▸(*b*)].

Under biological conditions, the band 3 protein is generally present in RBC membranes in the form of a dimer, corresponding to an elongated elliptical section with major/minor axes 105/31 Å (Xia *et al.*, 2022 ▸). The scattering from this type of structure can be calculated from the general construction in Fig. 2 ▸ by using cylinders with elliptical cross sections instead of circular. For the hard-disc model, this is done by replacing equation (14) by the form factor for ellipses, and by identifying the hard-disc radius in the structure factor in equation (15) with the ellipse’s major axis. For the Boolean model, this is done by replacing equation (18) by the geometric covariogram of randomly oriented ellipses. The results of the fits are reported in Fig. S1 and Table S1 in the supporting information. Globally, the quality of the fits is slightly degraded when dimers are assumed instead of monomers. However, the effect is very limited. This results from the convolution in the second term of equation (21), by which subtle features in protein scattering *I*_p_(*q*_*xy*_) are smeared in the total scattering pattern of the membrane. In the following, we assume band 3 monomers, modelled as cylinders with a simple circular section.

**Figure 7:**
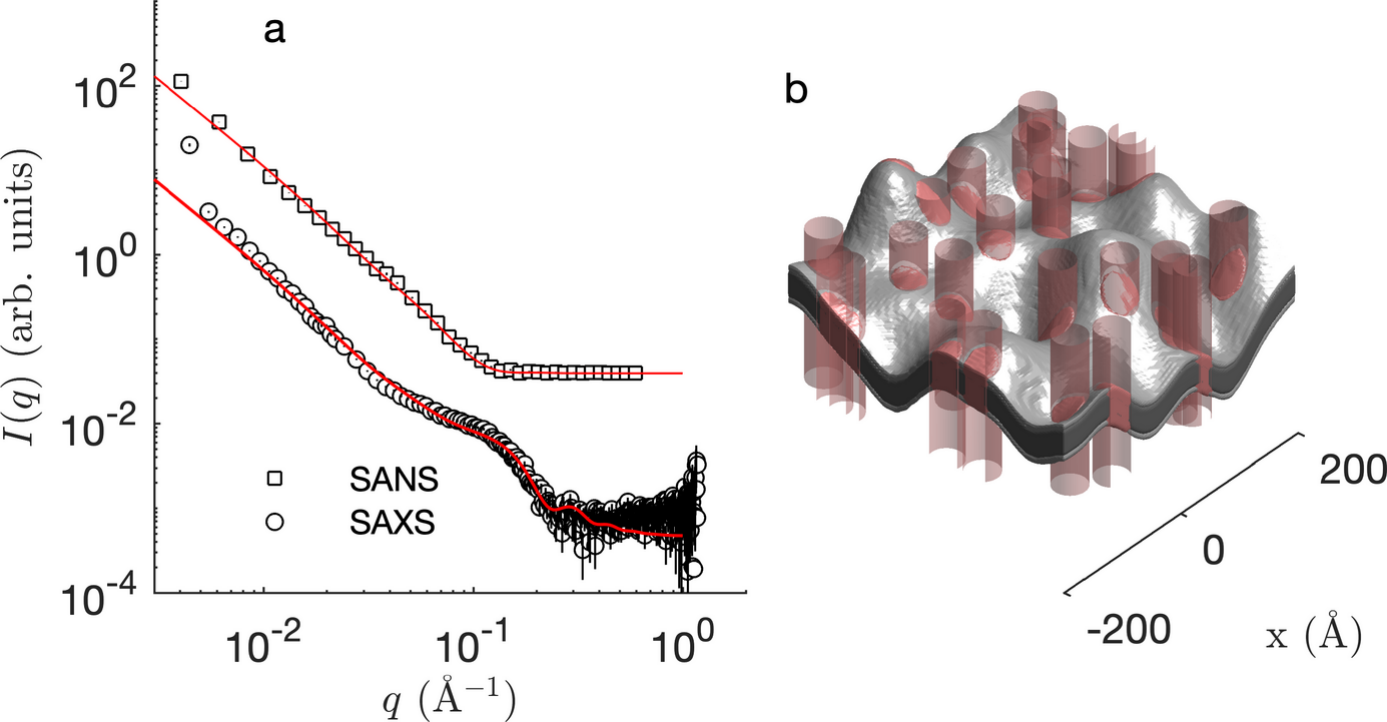
(*a*) Fitting of the membrane SANS and SAXS data with the Gaussian random membrane model with included band 3 protein. The circles are the data [same as in Fig. 1 ▸(*a*)] and the solid lines are the fits. (*b*) Particular realization of the model with the protein-defining cylinders shown in red, and with the two shades of grey highlighting the chain and head parts. The fitting range extends from *q* = 8 × 10^−3^ Å^−1^ to *q* = 0.5 Å^−1^.

As an alternative to the slab model, the same SAXS and SANS data sets were also jointly analysed with the Gaussian membrane model. The result of the fit is shown in Fig. 7 ▸, together with a realization of the model. The fitted parameters show that the membrane is subject to significant fluctuation. In particular, the standard deviation of the membrane displacement in direction *z* is *l*_α_ ≃ 23 Å and the lateral extension of the deformations in the membrane plane is *l*_*xy*_ ≃ 63 Å. These values are of the same order of magnitude as those reported in earlier work for different types of membranes (Monzel & Sengupta, 2016 ▸; Gommes *et al.*, 2024 ▸). Because of these fluctuations, the actual area of the membrane is slightly larger than its projected area in the *xy* plane. The roughness factor, calculated using equation (S10) of Gommes *et al.* (2024 ▸), is about 1.2. Using this value, the equivalent thicknesses of the chain and head parts of the membrane can be obtained from the fitted parameters of the Gaussian membrane model as the volume-to-area ratios. The values are *l*_C_ ≃ 16.1 Å and *l*_H_ ≃ 5 Å, corresponding to a total membrane thickness of 42.2 Å, very similar to the slab model.

These results agree with previous work on oriented multi-lamellar stacks of RBC membranes (Himbert *et al.*, 2017 ▸; Himbert & Rheinstädter, 2022 ▸). In that work liquid-ordered (l_o_) and -disordered (l_d_) lipid phases in RBC membranes, with thicknesses of 48 Å l_o_ and 41 Å l_d_, and a membrane protein fraction with a thickness of 40.6 Å were reported. Reported ratios of the components are 30.2% l_o_, 45.0% l_d_ and 24.8% membrane protein (Himbert *et al.*, 2017 ▸; Himbert & Rheinstädter, 2022 ▸). Taking into account the weighted fractions of the lipid ordered and disordered phases, an average thickness of the RBC membrane of 43.8 Å is obtained.

**Figure 8:**
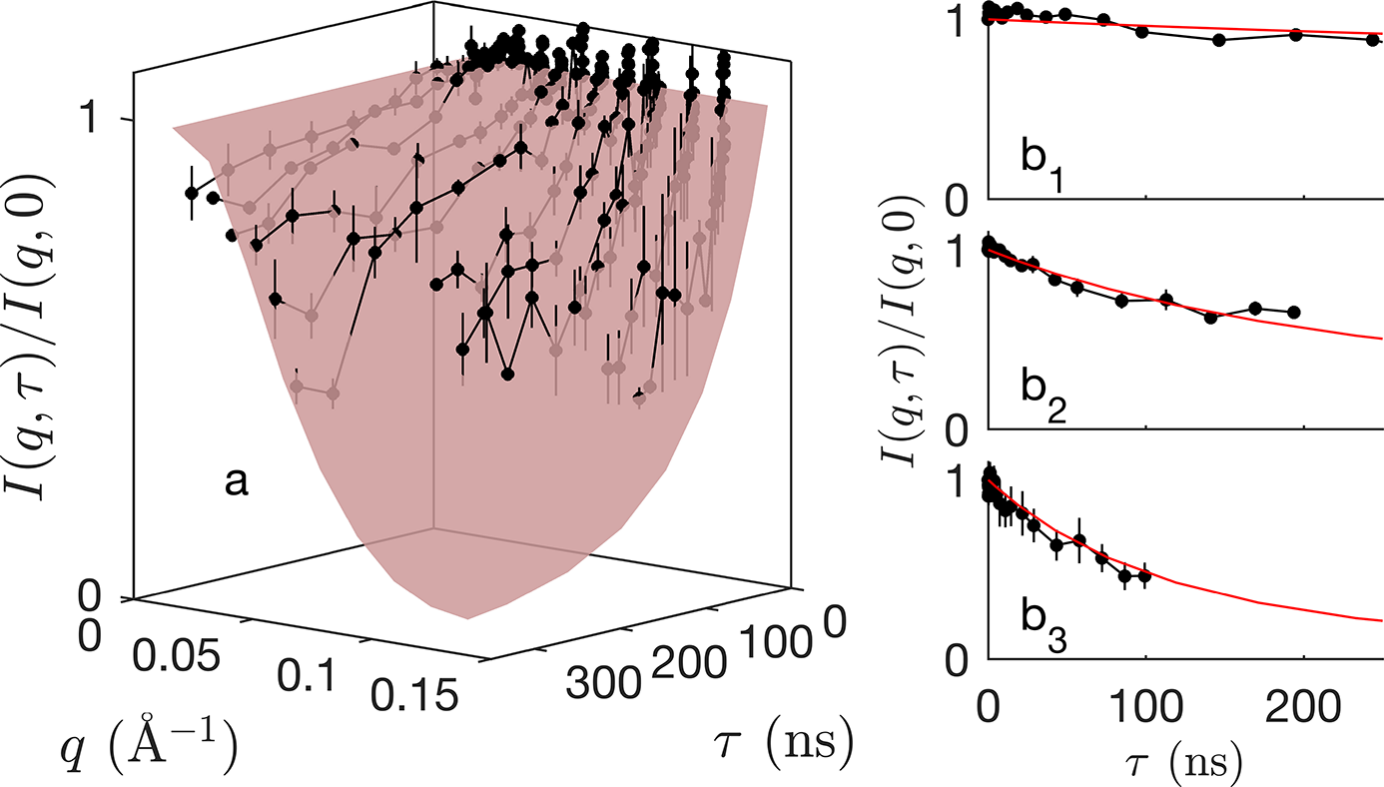
(*a*) One-parameter fit of the NSE data with the Gaussian membrane model [same data as in Fig. 1 ▸(*b*)]. The red surface is obtained with the same structural parameters as the SAXS and SANS data, with fitted diffusion coefficient *D* = 1.85 Å^2^ ns. (*b*) Highlighting the fit for (*b*_1_) *q* = 0.036 Å^−1^, (*b*_2_) *q* = 0.071 Å^−1^ and (*b*_3_) *q* = 0.109 Å^−1^.

Elastic scattering data, such as SAXS or SANS, capture only the instantaneous structure of the membrane and are blind to its dynamics. The realizations in Figs. 4 ▸(*b*)–4 ▸(*d*) and Fig. 7 ▸(*b*) are therefore to be understood as instantaneous snapshots of a dynamic structure. Insights into the dynamics of the fluctuations can be experimentally obtained from NSE experiments: the thus-obtained intermediate scattering function *I*(*q*, τ) is plotted in Fig. 8 ▸. Because the SLDs relevant to these experiments are the same as for SANS, one can safely assume that the band 3 protein does not contribute significantly to the NSE data [Fig. 6 ▸(*a*)]. In other words, the NSE data can be modelled on the basis of the first term in equation (9), namely 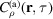, where the proteins only contribute through the modification of the membrane’s average SLD.

**Figure 9:**
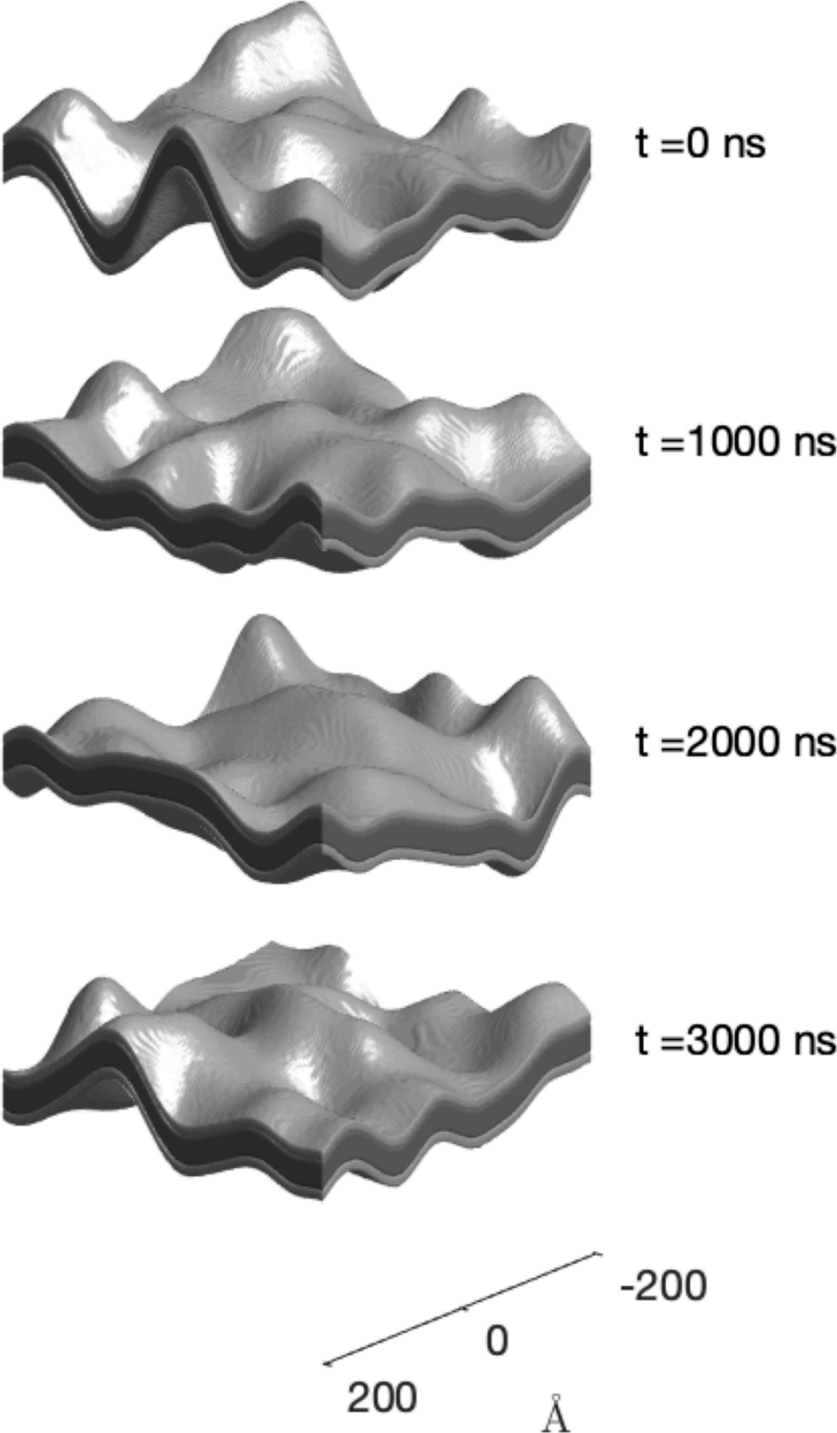
Time-dependent realization of the Gaussian random membrane model, with structural parameters obtained from the SAXS and SANS data and dynamic parameter *D* obtained from NSE data. The two shades of grey indicate the head and chain parts of the membrane.

The fitting of the Gaussian membrane model to the NSE data is illustrated in Fig. 8 ▸. All structural parameters of the model were fixed to their values obtained from the fitting of the SANS and SAXS data. The dynamics of the membrane are then described by the function *g*_*W*_(*r_xy_*, τ) from equation (28), which has the diffusion coefficient *D* as the only additional parameter. The value obtained from the least-squares fitting of the entire NSE data set is *D* ≃ 1.85 Å^2^ ns^−1^. This value can be converted to a typical correlation time by noting that any memory of the earlier structure is lost when the wave packets responsible for the membrane deformation have diffused over a distance larger than their size *l*_*xy*_. The correlation time is therefore 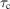 = 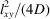 ≃ 960 ns, where the factor 4 is typical of 2D random walks. Time-dependent realizations of the model over a time span of 3τ_c_ are shown in Fig. 9 ▸.

## 5. Conclusions

We have developed a general mathematical construction for adding protein-like inclusions to any pre-existing membrane model and for calculating the resulting scattering cross section. The general construction is that of Fig. 2 ▸, where the inclusions are defined as cylinders that intersect with the membrane. The shape and size of the inclusions can be tuned via the cross section of the cylinders. Through suitable choice of the SLDs along the cylinder, the construction allows one to model inclusions that would cross an arbitrary fraction of the membrane and/or possibly protrude from it (Fig. 3 ▸).

In all cases, the elastic and/or inelastic scattering cross sections are calculated using the Fourier transform of equation (9). This expression contains two contributions calculated from the pre-existing membrane model, with two SLDs corresponding to the protein-dependent average and to the local contrast between the protein and the membrane. The protein structure is comprehensively captured through its space and time correlation function in the membrane plane. The versatility of the approach has been illustrated by applying it to two qualitatively different pre-existing membrane models, namely a slab model to analyse the SAXS and SANS of RBC membranes, and a Gaussian model to analyse their SAXS, SANS and NSE.

The present work has several ramifications. Higher-resolution versions of the model could easily be produced to describe *e.g.* channel proteins. This could be done by introducing two concentric cylinders in the construction of Fig. 2 ▸, for the inner channel and for the surrounding wall. On the experimental side, neutron scattering length contrast between the band 3 protein and the RBC membrane proved insufficient to track the band 3 mobility in the available NSE data. Should such experiments be possible with a suitable contrast, the models presented here would offer simple procedures to discriminate in the NSE data between the contribution from the membrane fluctuations and that from the protein mobility within the membrane.

## Supplementary Material

Additional figure and table. DOI: 10.1107/S1600576725007277/jo5125sup1.pdf

## Data Availability

Neutron scattering raw data can be downloaded from the ILL data repository https://doi.ill.fr/10.5291/ILL-DATA.8-03-1061 (Matsarskaia *et al.*, 2023 ▸).
