# Supplementary material for: Model for small-angle scattering analysis of membranes with protein-like inclusions
Source: J Appl Crystallogr. 2025 Sep 12;58(Pt 5):1571–81. doi: 10.1107/S1600576725007277 (PMC12502873; doi:10.1107/S1600576725007277)
Supplement: Supplementary file 1 [file j-58-01571-sup1.pdf]

JOURNAL OF
APPLIED
CRYSTALLOGRAPHY

Volume 58 (2025)

Supporting information for article:

Model for small-angle scattering analysis of membranes with protein-like inclusions

Cedric J. Gommes, Olga Matsarskaia, Julio M. Pusterla, Igor Graf von Westarp, Baohu Wu, Orsolya Czakkel and Andreas M. Stadler

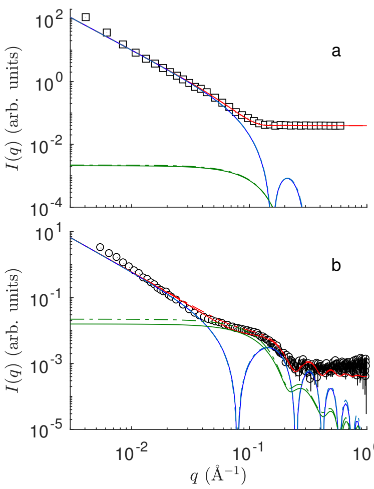

(a) Band 3 monomer, disk

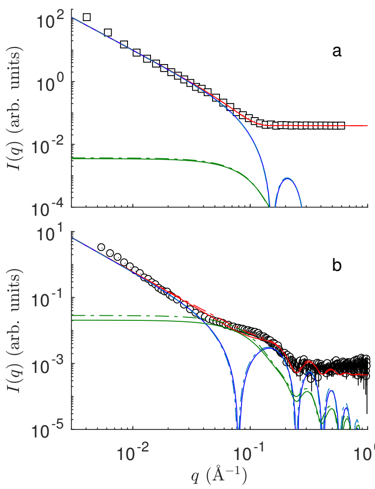

(b) Band 3 dimer, disk

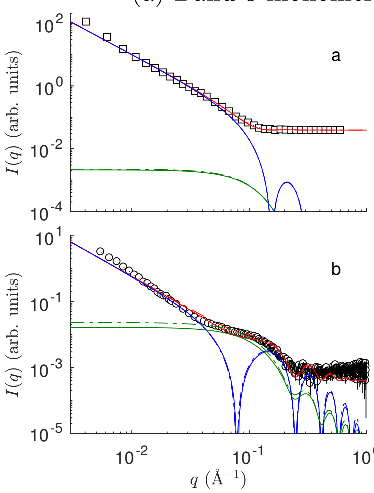

(c) Band 3 monomer, ellipse

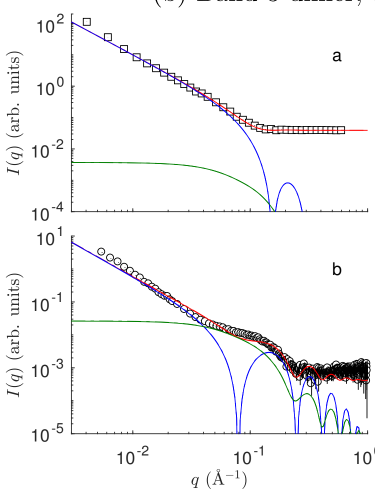

(d) Band 3 dimer, ellipse

Figure SI–1: Fits of the SANS (a) and SAXS (b) data, with various models for the band 3 equivalent cylinder section, namely: (a) disk with radius $R_P = 21.4$ Å (same as in the main text), (b) disk with radius $R_P = 28.5$ Å, (c) ellipse with half axes $a_P = 29.5$ Å and $b_P = 15.5$ Å, (d) ellipse with half axes $a_P = 52.5$ Å and $b_P = 15.5$ Å.

			a_P (Å)	b_P (Å)	l_C (Å)	l_H (Å)	χ^2 (-)
Disks	Monomer	Boolean	21.4	21.4	16.3	5.0	0.35
		Hard-disk	21.4	21.4	16.6	4.5	0.35
	Dimer	Boolean	28.4	28.4	16.0	5.6	0.45
		Hard-disk	28.4	28.4	16.6	5.1	0.42
Ellipses	Monomer	Boolean	29.5	15.5	16.4	5.0	0.38
		Hard-disk	29.5	15.5	16.8	4.5	0.43
	Dimer	Boolean	52.5	15.5	16.3	5.1	0.49
		Hard-disk	52.5	15.5	16.6	5.1	0.37

Table SI–1: Parameters of the various models of the band 3 proteins: a_P, b_P: major and minor axes of the ellipse (imposed); l_C, l_H: lengths of the hydrophobic chain and hydrophylic heads of the bilayer (fitted); χ^2: squared sum of errors (logarithmic).
